# Remimazolam versus propofol on seizure adequacy in electroconvulsive therapy: a retrospective cohort study

**DOI:** 10.3389/fpsyt.2026.1885189

**Published:** 2026-07-21

**Authors:** Haibo Song, Xingxing Yin, Feng Wang, Yongming Zhang, Menglu Li, Bo Li, Mingyang Zhou, Yiying Li, Xiangjie Li, Xiufang Shi, Tianqi Shen

**Affiliations:** 1 Department of Anesthesiology, No. 984 hospital of the PLA, Beijing, China; 2 Combat Trauma Center, No. 984 hospital of the PLA, Beijing, China; 3 Department of Psychiatry and Psychology, No. 984 hospital of the PLA, Beijing, China

**Keywords:** electroconvulsive therapy, mental disorders, propofol, remimazolam, seizure

## Abstract

**Objective:**

Electroconvulsive therapy (ECT) is a core treatment modality for severe mental disorders, and the choice of anesthetic induction agent directly impacts seizure quality and therapeutic outcomes. This study aimed to compare the effects of remimazolam versus propofol on seizure adequacy during ECT.

**Methods:**

This retrospective secondary analysis was conducted using data from a prospective observational cohort. A total of 859 ECT sessions from 114 patients were included. The primary outcome was the rate of adequate seizure, defined as an electroencephalographic (EEG) ictal duration of ≥15 seconds. Secondary outcomes included EEG seizure duration, post-ictal suppression index (PSI), maximum sustained power (MSP), and average seizure energy index (ASEI). Stabilized inverse probability of treatment weighting was employed to balance baseline covariates between groups, with balance assessed by standardized mean differences. Generalized linear mixed models were used to compare intergroup differences, accounting for random effects at the patient level. Subgroup analysis, E-value calculation, and propensity score matching (PSM) at 1:1 and 1:2 ratios were performed as sensitivity analyses to assess the robustness of the results.

**Results:**

A total of 859 ECT sessions were included, with 179 in the remimazolam group and 680 in the propofol group. Baseline characteristics were well-balanced between groups after weighting. The remimazolam group exhibited a significantly higher rate of adequate seizures compared with the propofol group (adjusted odds ratio: 12.054, 95% confidence interval: 5.758–25.233, *P* < 0.001), along with prolonged EEG seizure duration and elevated MSP. No significant between-group differences were observed in PSI or ASEI. The results of all sensitivity analyses were consistent with those of the primary analysis.

**Conclusion:**

Remimazolam was associated with a higher rate of EEG seizure adequacy and longer EEG seizure duration; however, whether this electrophysiological advantage translates into better clinical outcomes remains to be investigated in prospective studies.

## 1 Introduction

The incidence of mental disorders has been increasing year by year. Currently, mental disorders remain one of the top ten leading causes of global disease burden, with no downward trend in burden levels (1). Mental disorders not only impair health but also significantly increase suicide risk and associated mortality (2–4). At present, conventional pharmacotherapy for various mental disorders has limited efficacy, long onset times, and relatively low remission and response rates (5).

Electroconvulsive therapy (ECT) induces controlled seizure activity through electrical stimulation while the patient is in a state of muscle relaxation and sedation achieved via anesthetic techniques, thereby modulating brain function (6). It is particularly indicated for patients with severe mental disorders accompanied by intense suicidal ideation, catatonic stupor, or refusal of food, enabling rapid symptom control (7), and represents one of the important therapeutic modalities for psychiatric disorders (8, 9).

ECT treatment outcomes are influenced by multiple factors (10, 11), among which the selection of optimal anesthetic agents constitutes a major challenge in ECT (12). The commonly used combination in China is propofol with succinylcholine; however, propofol elevates seizure threshold and shortens seizure duration (13), potentially exerting adverse effects on ECT efficacy. Given the inhibitory effect of propofol on seizure activity, exploring alternative agents with minimal impact on seizure duration is of particular significance.

Remimazolam is a novel benzodiazepine characterized by ultra-short onset and recovery profiles, and its effects can be rapidly reversed by flumazenil (14). Its favorable pharmacokinetic properties and minimal influence on seizure duration (15) have supported its safe use in sedation for brief examinations and procedures such as gastrointestinal endoscopy (16), also rendering it a potential candidate for ECT anesthesia.

This study aims to compare the effects of remimazolam versus propofol on ECT seizure adequacy, thereby providing novel evidence for anesthetic agent selection in ECT.

## 2 Materials and methods

### 2.1 Study design

This study represents a secondary analysis of a prospective observational study. The original study enrolled patients who underwent electroconvulsive therapy at the No. 984 hospital of the PLA between August 2025 and February 2026. Both the original study and the present study were approved by the Ethics Committee of the No. 984 hospital of the PLA (No. 2025060512; No. 2026050701). Informed consent was waived owing to the retrospective nature of this study. This report adheres to the Strengthening the Reporting of Observational Studies in Epidemiology (STROBE) statement (17).

### 2.2 ECT procedure and anesthesia management protocol

All patients fasted from food and water for 8 hours preoperatively, and emergency airway equipment and a defibrillator were prepared. Upon entering the treatment room, heart rate, non-invasive blood pressure, and pulse oxygen saturation were continuously monitored until the patient returned to the ward. Electroconvulsive therapy was administered using a Thymatron System IV (Somatics LLC, Lake Bluff, IL, USA), with simultaneous recording of electrocardiogram and electroencephalogram signals. All treatments employed a bilateral bitemporal electrode placement. Routine hyperventilation was not performed prior to stimulation (10); each patient received a tidal volume of 6–8 ml·kg^-1^ and a respiratory rate of 10–15 breaths/min. After intravenous injection of atropine 0.5 mg, propofol 1.5–2 mg·kg^-1^ or remimazolam 0.15–0.2 mg·kg^-1^ was slowly injected until the eyelash reflex disappeared (18, 19); subsequently, succinylcholine 0.8–1 mg·kg^-1^ was administered intravenously with assisted mask ventilation. After adequate muscle relaxation was achieved, a bite block was inserted and electrical stimulation was delivered. Upon recovery of spontaneous breathing, patients were transferred to the post-anesthesia care unit; they were returned to the ward after full awakening and an Aldrete score ≥9.

In the original study, the choice of anesthetic regimen was determined by the routine clinical practice of the attending anesthesiologist on duty, who was assigned according to a fixed shift schedule. ECT was typically delivered in LOW 0.5 mode, with an initial stimulus dose of (age – 5)%, and subsequent stimulus doses were adjusted by the psychiatrist based on the previous seizure response and the patient’s clinical presentation.

### 2.3 Study population

The inclusion criteria for this study were: 1. patients with psychiatric disorders undergoing ECT; 2. anesthetic induction with propofol or remimazolam. The exclusion criteria were: 1. patients with temporary ECT cancellation due to inadequate nil per os duration or fever; 2. those with missing post-treatment electroencephalogram (EEG) results.

A single ECT session served as the unit of analysis. Based on the anesthetic induction agent actually administered during each session, the included treatment sessions were categorized as: 1. remimazolam group: ECT sessions using remimazolam as the induction agent; 2. propofol group: ECT sessions using propofol as the induction agent. The same patient could receive multiple treatment sessions and be included in different drug groups; each session was independently assigned according to the actual agent administered.

### 2.4 Observed variables and covariates

The primary outcome was the success rate of adequate post-ECT seizure, defined as electroencephalographic seizure duration ≥ 15 s (20). Secondary outcomes included: 1. Electroencephalographic seizure duration; 2. Postictal suppression index (PSI); 3. Maximum sustained power (MSP); 4. Average seizure energy index (ASEI).

This study employed a generalized linear mixed model (GLMM) with patient as a random intercept to account for within-subject correlation (21). Stabilized inverse probability of treatment weighting (sIPTW) was applied to balance treatment-related covariates (22), including: concomitant medications on the day prior to treatment (Antidepressants, Antihistamines, Anxiolytics, Antipsychotics, Benzodiazepines, Sedative-hypnotics, Mood stabilizers, Monoamine oxidase inhibitors, and Adjunctive medications), whether the session was the First ECT within a cycle, and Stimulus charge.

### 2.5 Statistical analysis

To address covariate imbalance between the two treatment groups, sIPTW was employed to construct a weighted cohort. Treatment drug assignment served as the dependent variable, with the aforementioned treatment-related covariates as independent variables. Propensity scores were estimated via logistic regression, and stabilized weights were calculated. Standardized mean difference (SMD) was used to assess intergroup covariate balance before and after sIPTW, with an absolute SMD < 0.10 as the criterion for covariate balance. GLMM was applied to analyze the weighted cohort to estimate treatment effects. For baseline characteristics, continuous variables were subjected to Kolmogorov-Smirnov normality testing; those conforming to normal distribution were expressed as mean ± standard deviation, and non-normally distributed variables as median (interquartile range). Categorical variables were expressed as frequency (percentage).

The primary outcome was a binary variable, analyzed using GLMM binary logistic regression, with results expressed as adjusted odds ratio (aOR) and 95% confidence interval (95%CI). Secondary outcomes were continuous variables, analyzed using GLMM linear regression, with results expressed as adjusted mean difference (aMD) and 95%CI. Subgroup analysis and E-value (23) were employed to assess the robustness of the primary outcome. Sensitivity analyses were performed using PSM at 1:1 and 1:2 matching ratios, respectively. These analyses were not intended to replace the primary sIPTW analysis that retained the full analytic sample, but rather to assess whether the direction and magnitude of the primary association remained robust under a matched-sample design (24, 25). All statistical analyses and graphical work were performed using R version 4.4.2. Statistical significance was defined as two-sided *P* < 0.05.

## 3 Results

### 3.1 General characteristics

**Figure 1:**
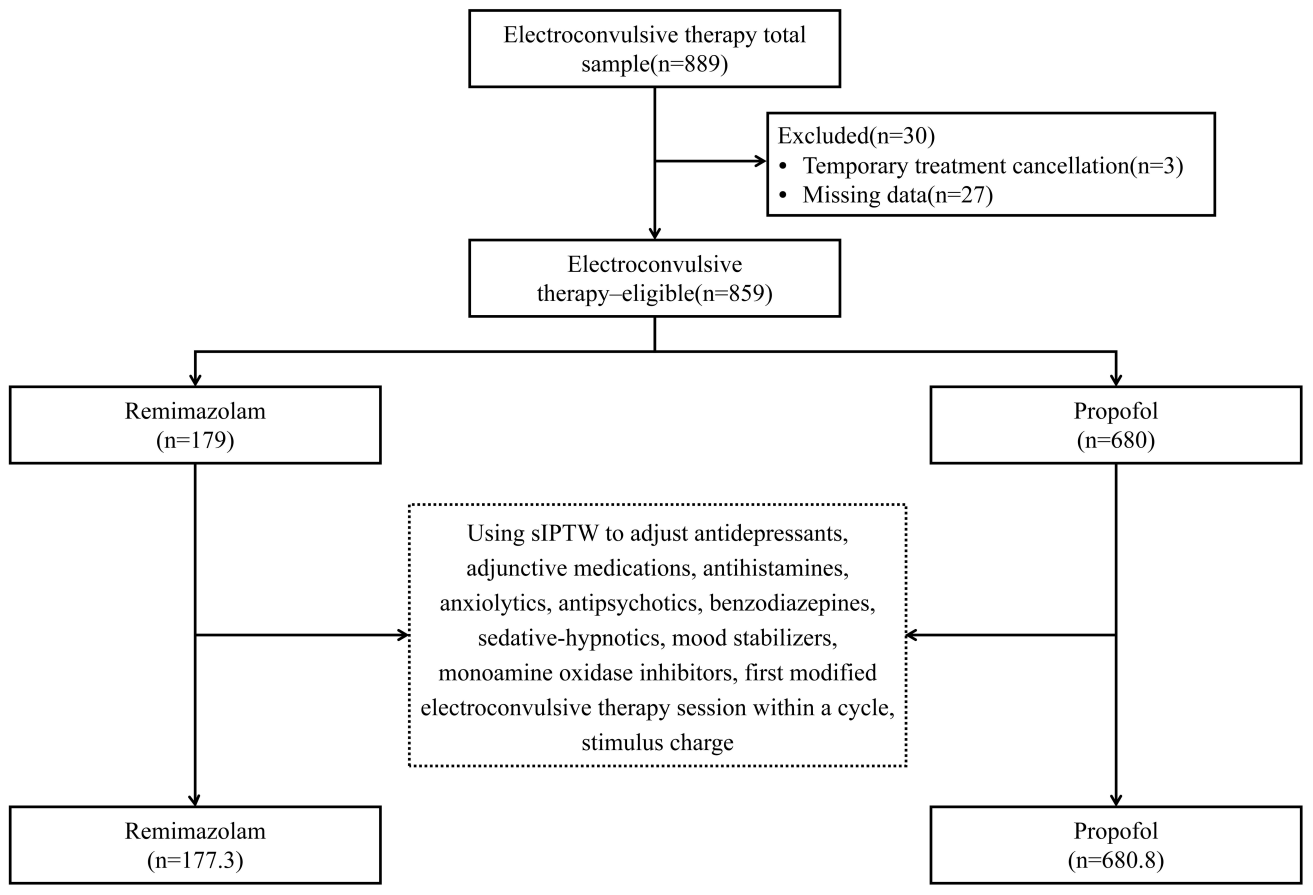
Screening flowchart of study participants. sIPTW, Stabilized Inverse Probability of Treatment Weighting.

According to the inclusion and exclusion criteria, 889 ECT sessions performed in 116 patients met the inclusion criteria. Based on the exclusion criteria, 859 ECT sessions in 114 patients were included in the analysis, among which 680 sessions used propofol as the anesthetic induction agent and 179 sessions used remimazolam induction (**Figure 1**). Among the 114 enrolled patients, the median age was 26.0 (22.0, 38.0) years, body weight was 70.0 (62.0, 80.0) kg, 73 (64.0%) were male, 36.0% (41 patients) were married, and 50 patients (43.9%) were diagnosed with schizophrenia. Regarding medical history, 41 patients (36.0%) had a smoking history, 5 patients (4.4%) had comorbid hypertension, and 6 patients (5.3%) had comorbid diabetes (**Supplementary Table 1**).

### 3.2 Weighted cohort

The baseline characteristics of the two groups before and after weighting are presented in **Table 1**. In the original cohort, the remimazolam and propofol groups exhibited imbalance in preoperative adjunctive medication use (22.9% vs. 9.1%, SMD = 0.383), preoperative antidepressant use (57.5% vs. 52.4%, SMD = 0.104), antipsychotic use (74.3% vs. 67.2%, SMD = 0.156), antihistamine use (6.7% vs. 4.0%, SMD = 0.122), anxiolytic use (26.8% vs. 21.3%, SMD = 0.129), benzodiazepine use (16.8% vs. 10.3%, SMD = 0.190), and monoamine oxidase inhibitor use (19.0% vs. 23.8%, SMD = 0.118). Regarding treatment parameters, intergroup difference existed in intraoperative stimulus charge (150.3 vs. 144.8 mC, SMD = 0.149). The two groups were generally comparable in preoperative sedative-hypnotic use (6.1% vs. 4.0%, SMD = 0.099), mood stabilizer use (5.6% vs. 5.0%, SMD = 0.026), and proportion of first-session treatments (11.7% vs. 13.8%, SMD = 0.063). Following sIPTW weighting, the SMD for all covariates decreased below 0.10, and the weighted cohort achieved satisfactory intergroup balance; intergroup differences before and after weighting are detailed in **Table 1**.

**Table 1:** Baseline characteristics before and after sIPTW.

Variable (n, %)/m(IQR)	Before sIPTW				After sIPTW			
	Total n = 859	Remimazolam n = 179	Propofol n = 680	SMD	Total n = 858.1	Remimazolam n = 177.3	Propofol n = 680.8	SMD
Premedication								
Antidepressants	459(53.4)	103(57.5)	356(52.4)	0.104	414.2(48.3)	100.7(56.8)	366.7(53.9)	0.060
Adjunctive medications	103(12.0)	41 (22.9)	62 (9.1)	0.383	104.6(12.2)	22.0 (12.4)	82.6 (12.1)	0.008
Antihistamines	39(4.5)	12 (6.7)	27 (4.0)	0.122	37.0(4.3)	6.8 (3.8)	30.2(4.4)	0.032
Anxiolytics	193(22.5)	48 (26.8)	145 (21.3)	0.129	197.1(23.0)	42.8 (24.1)	154.3 (22.7)	0.034
Antipsychotics	590(68.7)	133 (74.3)	457 (67.2)	0.156	592.2(69.0)	123.9 (69.9)	468.3 (68.8)	0.024
Benzodiazepines	100(11.6)	30 (16.8)	70 (10.3)	0.190	101.9(11.9)	21.8 (12.3)	80.1 (11.8)	0.016
Sedative-hypnotics	38(4.4)	11 (6.1)	27 (4.0)	0.099	39.1(4.6)	8.5 (4.8)	30.6 (4.5)	0.015
Mood stabilizers	44(5.1)	10 (5.6)	34 (5.0)	0.026	41.5(4.8)	7.1 (4.0)	34.4 (5.1)	0.050
Monoamine oxidase inhibitors	196(22.8)	34 (19.0)	162 (23.8)	0.118	192.7(22.5)	38.1 (21.5)	154.6 (22.7)	0.030
Treatment status								
First ECT session within a cycle	115(13.4)	21 (11.7)	94 (13.8)	0.063	117.9(13.7)	25.8 (14.5)	92.1 (13.5)	0.029
Stimulus charge (mC)	146.7 (105.8, 204.2)	150.3 (111.2, 227.2)	144.8 (105.2, 203.0)	0.149	146.5 (105.8, 204.1)	142.1 (106.1, 202.6)	148.9 (105.7, 204.7)	0.017

sIPTW, Stabilized inverse probability of treatment weighting; IQR, Interquartile range; SMD, Standardized mean difference; ECT, Electroconvulsive therapy.

### 3.3 Effect of remimazolam on seizure adequacy

**Figure 2:**
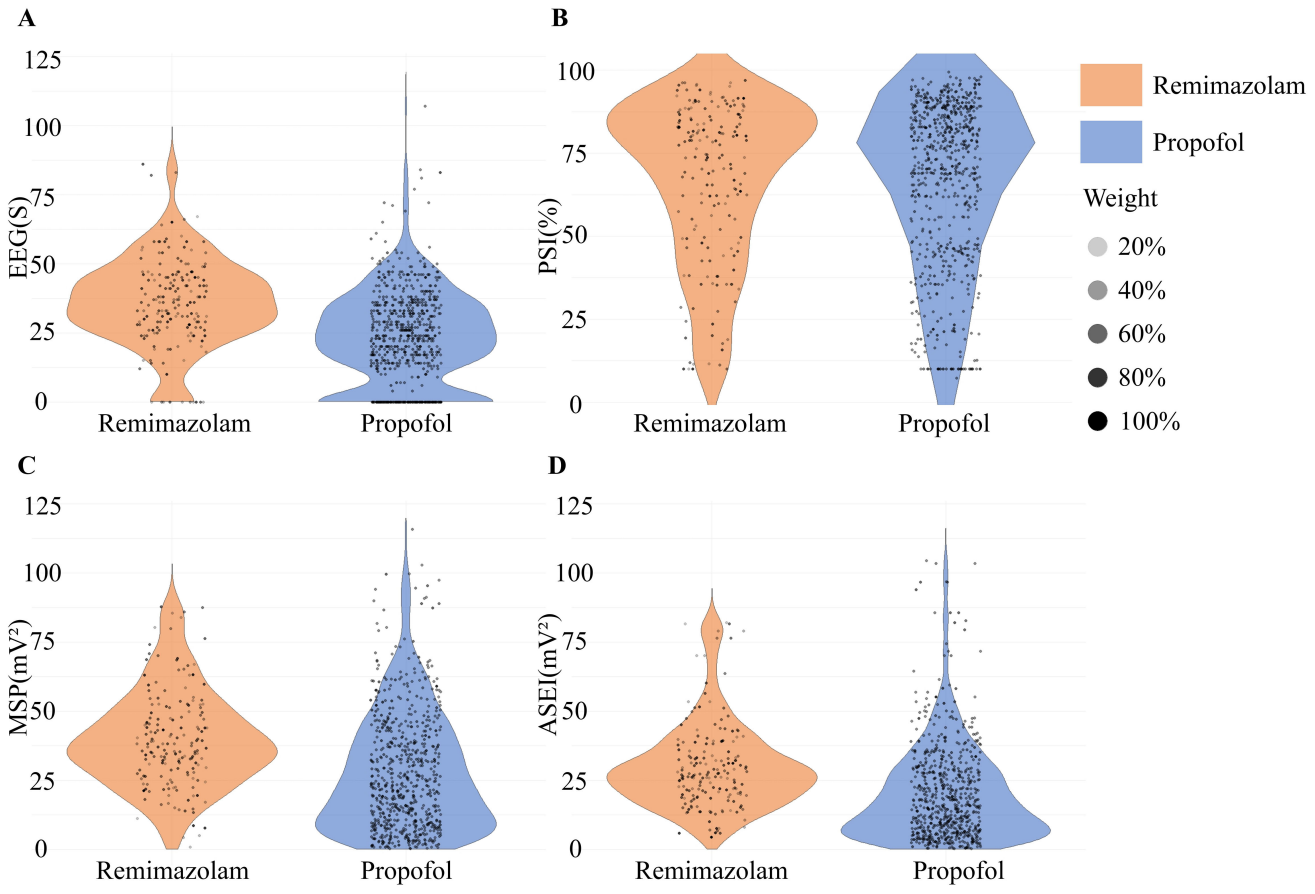
Comparison of ECT electroencephalographic monitoring parameters between the remimazolam and propofol groups. **(A)** Electroencephalographic sustained seizure duration; **(B)** Postictal suppression index; **(C)** Maximum sustained power; **(D)** Average seizure energy index. In the violin plots, each point represents one ECT session, with point shading reflecting sIPTW weight.

In the original cohort, the remimazolam group demonstrated a higher adequate seizure rate than the propofol group (91.6% vs. 69.2%). In the weighted cohort with adjustment for stimulus charge and first ECT session as covariates, the remimazolam group continued to show a significantly higher adequate seizure rate compared with the propofol group (aOR = 12.054, 95% CI: 5.758–25.233, *P* < 0.001). The remimazolam group exhibited longer electroencephalographic seizure duration and higher MSP than the propofol group (EEG seizure duration: aMD = 17.082 s, 95% CI: 14.382–19.781, *P* < 0.001; MSP: aMD = 10.440 mV², 95% CI: 6.323–14.557, *P* < 0.001), whereas no significant differences were observed between the two groups in PSI or ASEI (PSI: aMD = −0.320, 95% CI: −1.025–0.385, *P* = 0.374; ASEI: aMD = 2.718 mV², 95% CI: −0.140–5.576, *P* = 0.063). See **Figure 2** and **Table 2** for details.

**Table 2:** Comparison of outcome indicators between the two groups.

Outcome variable (n, %)/M(IQR)	Remimazolam n = 177.3	Propofol n = 680.8	aOR/aMD (95% CI)	*P* value
EEG adequate seizure rate	163.9(92.5)	466.6(68.5)	12.054(5.758-25.233)	< 0.001
EEG seizure duration (s)	36.1(28.0, 46.0)	23.0(10.0, 35.0)	17.082(14.382-19.781)	< 0.001
PSI (%)	74.4(49.8,85.1)	75.4(47.5,87.4)	-0.320(-1.025-0.385)	0.374
MSP (mV²)	38.3(29.3, 51.8)	23.7(10.3, 42.7)	10.440 (6.323-14.557)	< 0.001
ASEI (mV²)	27.0(19.4, 36.3)	16.0(7.1, 29.0)	2.718(-0.140-5.576)	0.063

IQR, Interquartile range; aOR, Adjusted odds ratio; aMD, Adjusted mean Difference; PSI, Postictal suppression index; MSP, Maximum sustained power; ASEI, Average seizure energy inde.

### 3.4 Result stability

**Figure 3:**
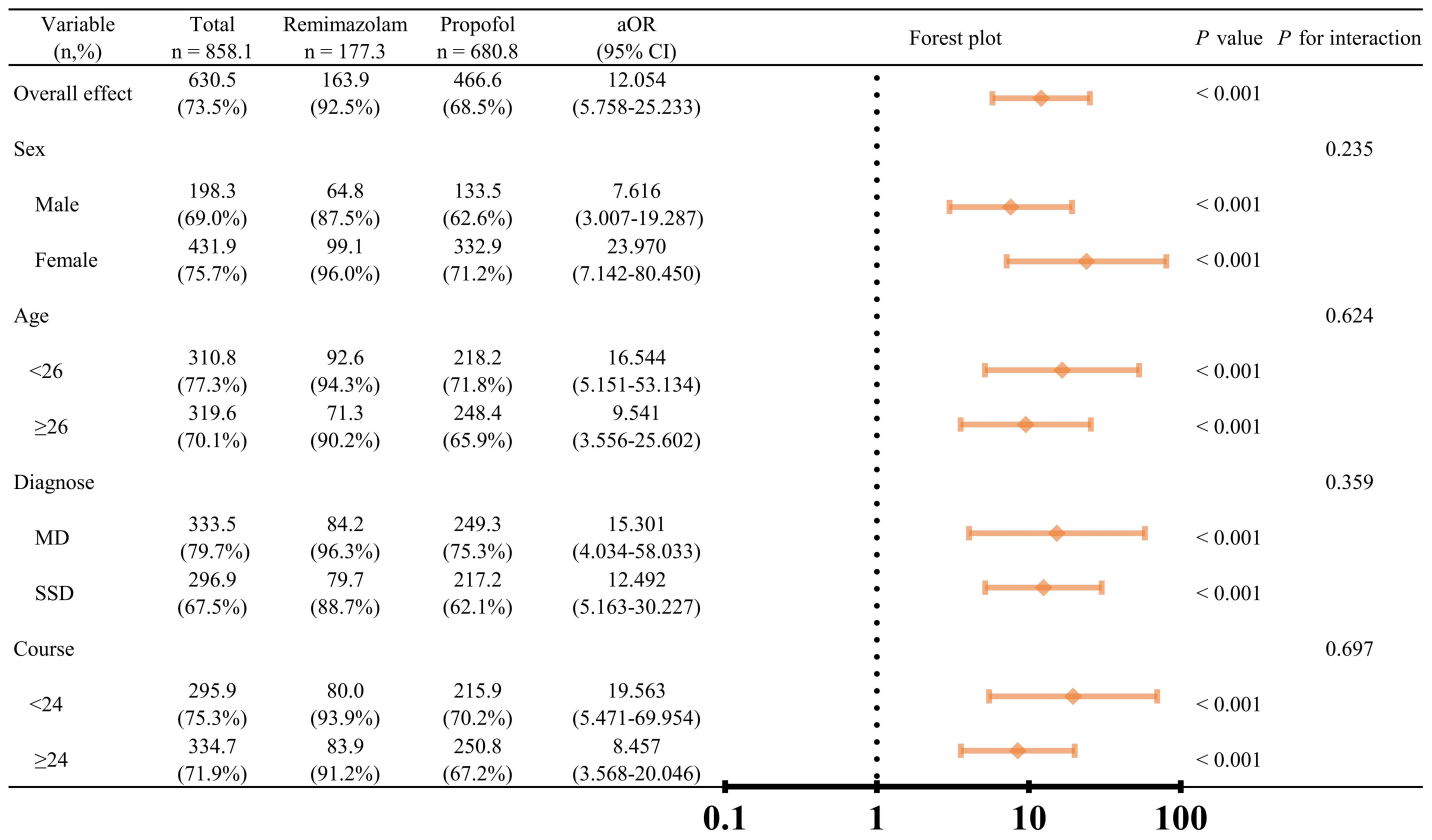
Forest plot of subgroup analysis of the effect of remimazolam versus propofol on electroencephalographic adequate seizure rate. The left table presents the weighted sample size, adjusted odds ratio (aOR), and 95% confidence interval (95% CI) for each subgroup; the right forest plot displays the aOR and 95% CI for each subgroup, with the vertical dashed line indicating the null line (OR = 1). OR, Odds Ratio; 95%CI, 95% Confidence Limits.

We conducted subgroup analyses stratified by sex, age, disease type, and disease duration. The results demonstrated that the remimazolam group consistently exhibited a significantly higher adequate seizure rate than the propofol group across all subgroups (*P* < 0.001), with no statistically significant heterogeneity in intergroup differences (*P* > 0.05), thereby validating the cross-subgroup consistency of remimazolam’s advantage in improving post-ECT electroencephalographic adequate seizure rates (**Figure 3**). We employed E-value to assess the potential impact of unmeasured confounders on the outcome. The results indicated that an unobserved variable would require an OR greater than 6.401 to alter the direction of the findings, reflecting a certain degree of outcome robustness.

The PSM sensitivity analysis results were consistent with those of the primary sIPTW analysis. The remimazolam group showed a higher rate of seizure adequacy than the propofol group in both the 1:1 matched cohort and the 1:2 matched cohort (1:1 matched cohort: aOR = 15.365, 95% CI: 6.387–36.962, P < 0.001; 1:2 matched cohort: aOR = 13.173, 95% CI: 6.217–27.919, P < 0.001) (**Supplementary Figure 1**). These findings further confirmed the robustness of the association between remimazolam and a higher rate of EEG seizure adequacy.

## 4 Discussion

This study was a secondary analysis of data from a prospective observational cohort. Compared with propofol, the remimazolam group showed a higher rate of EEG seizure adequacy, longer EEG seizure duration, and higher MSP, but no significant difference in PSI was observed between the two groups. These findings suggest that the differences between the two agents may primarily lie in the ictal phase rather than in the postictal suppression period following seizure termination. Remimazolam may be more favorable for sustaining the duration and intensity of ictal EEG activity, but did not demonstrate an enhanced effect on postictal suppression, implying that its impact on ECT EEG seizure parameters may not be comprehensive.

The results of this study showed that the aOR for the rate of EEG seizure adequacy in the remimazolam group versus the propofol group were relatively large. We believe this phenomenon is primarily attributable to the strong inhibitory effect of propofol on seizures, rather than suggesting that remimazolam possesses an abnormal proconvulsant effect. In the propofol group, the rate of EEG seizure adequacy was only 68.5%, far lower than the 92.5% observed in the remimazolam group. This difference is also consistent with the clinical efficacy of the two agents: in clinical practice, propofol has been recommended for benzodiazepine-refractory status epilepticus (26, 27), further corroborating its pronounced seizure-suppressing capability. Therefore, the current large aOR values are more appropriately interpreted as a reflection of propofol’s potent antiepileptic effect.

The differences in outcomes between the two agents may stem from their distinct binding sites on the GABAA receptor, modes of action, and molecular pharmacological profiles. Previous studies have shown (28) that propofol acts on the GABAA receptor to produce GABA-independent neuronal hyperpolarization (29), which at the network level manifests as synchronized inhibition, leading to burst suppression—a state of profound central depression. This inhibition may, to some extent, increase the seizure threshold or cause evoked epileptic activity to terminate earlier, thereby shortening EEG seizure duration.

Remimazolam is a benzodiazepine that enhances chloride channel opening through modulation of the GABAA receptor (30, 31). This property produces a seizure-suppressive effect distinct from that of propofol: its depth of inhibition is naturally constrained by local GABA levels, making burst suppression less likely to occur. This pharmacological difference may help explain the longer EEG seizure duration observed in the remimazolam group in the present study (32). However, the underlying mechanisms still require further investigation and validation.

However, this study has several limitations. First, since the data were derived from real-world clinical practice, although we employed sIPTW, GLMM, and PSM sensitivity analyses to balance relevant covariates and account for the correlation arising from multiple treatments per patient, these methods cannot completely rule out the influence of unmeasured confounding and confounding by indication. Second, the follow-up duration was relatively short, precluding assessment of differences between remimazolam and propofol in long-term clinical efficacy and cognitive impairment. Finally, according to observational study reporting guidelines (17), outcome variables should have clearly defined data sources, measurement methods, and definitions. Although routine perioperative monitoring data were available in this study, safety endpoints were not prespecified study outcomes, and adverse events were not defined or systematically collected using standardized criteria. Therefore, this study does not allow a rigorous comparison of the safety profiles of remimazolam and propofol in ECT.

The central contribution of this study lies in its descriptive finding: remimazolam demonstrates a higher adequate seizure rate compared with propofol during ECT; however, large-scale prospective randomized controlled trials are required to validate this causal relationship. At the current evidence level, the characteristics of higher adequate seizure rates and prolonged seizure duration associated with remimazolam may serve as reference information for anesthetic agent selection in ECT, yet the ultimate decision should integrate individual patient characteristics, institutional practice conditions, and clinician clinical experience.

## Data Availability

The raw data supporting the conclusions of this article will be made available by the authors, without undue reservation.
