# Supplementary material for: Remimazolam versus propofol on seizure adequacy in electroconvulsive therapy: a retrospective cohort study
Source: Front Psychiatry. 2026 Jul 21;17:1885189. doi: 10.3389/fpsyt.2026.1885189 (PMC13433366; doi:10.3389/fpsyt.2026.1885189)
Supplement: Supplementary file 1 [file Table1.docx]

Table S 1 demographic characteristics of the enrolled patients

| Variable | Value |
| --- | --- |
| Age (years),median (IQR) | 26.0(22.0,38.0) |
| Weight (kg),median (IQR) | 70.0(62.0,80.0) |
| Sex,Male,n(%) | 73(64.0%) |
| Marital Status,n(%) | |
| Married | 41(36.0%) |
| Unmarried | 64(56.1%) |
| Divorced | 9(7.9%) |
| Diagnosis,n(%) | |
| Schizophrenia | 50(43.9%) |
| Mood Disorde | 64(56.1%) |
| Smoking history,n(%) | 41(36.0%) |
| Comorbid hypertension,n(%) | 5(4.4%) |
| Comorbid diabetes,n(%) | 6(5.3%) |

IQR: Interquartile range
